# Supplementary material for: MODY is prevalent in later-onset diabetes, has potential for targeted therapy but is challenging to identify
Source: Diabetes. Author manuscript; Available in PMC 2026 Jan 17. (PMC7618620; doi:10.2337/db25-0545)
Supplement: Supplementary Materials [file EMS210954-supplement-Supplementary_Materials.pdf]

Supplementary Materials

Supplementary Table 1: Clinical characteristics of individuals diagnosed with diabetes after age 40yrs in the UK Biobank and MyCode population.

Clinical Features	UK Biobank	MyCode
**N**	25,012	26,607
**Age at recruitment, y**	62.2 (56.8-66.1) (Missing n = 0)	63.9 (56.2-71.3) (Missing n = 0)
**Age at diagnosis, y**	56.5 (50.5-61.5) (Missing n = 0)	57.9 (50.7-65.5) (Missing n = 0)
**Females**	9,389 (37.5%) (Missing n = 0)	13,489 (50.7) (Missing n = 0)
**European Ancestry**	21,107 (84.4%) (Missing n = 0)	25,073 (94.2) (Missing n = 0)
**Parent with diabetes**	8,256 (33.0%) (Missing n = 916)	10,293 (38.7) (Missing n = 1900)
**BMI, Kg/m^2**	30.9 (27.7-34.8) (Missing n = 215)	33.6 (29.1-38.6) (Missing n = 180)

N (%) for categorical data and median (interquartile range) for the continuous data.

Supplemental Table 2: Pathogenic variants from the UK Biobank identified in this study.

Genomic Change	Gene	Protein Change	Nucleotide Change	Classification
11:17397788:C:T	*ABCC8*	p.Gly1256Ser	ENST00000302539.9:c.3766G>A	Likely Pathogenic
11:17460529:C:T	*ABCC8*	p.Val324Met	ENST00000302539.9:c.970G>A	Pathogenic
11:17404525:G:A	*ABCC8*	p.Arg1183Trp	ENST00000302539.9:c.3547C>T	Pathogenic
11:17413396:G:A	*ABCC8*	p.Arg826Trp	ENST00000302539.9:c.2476C>T	Pathogenic
11:17460582:C:T	*ABCC8*	p.Arg306His	ENST00000302539.9:c.917G>A	Pathogenic
7:44149977:G:A	*GCK*	p.Arg191Trp	ENST00000403799.8:c.571C>T	Pathogenic
7:44151069:C:T	*GCK*	p.Asp124Asn	ENST00000403799.8:c.370G>A	Pathogenic
7:44150004:C:T	*GCK*	p.Val182Met	ENST00000403799.8:c.544G>A	Pathogenic
7:44149797:G:GT	*GCK*	p.Tyr214*	ENST00000403799.8:c.641dup	Pathogenic
7:44149860:C:T	*GCK*	p.?	ENST00000403799.8:c.580-1G>A	Pathogenic
7:44153326:G:T	*GCK*	p.Tyr61*	ENST00000403799.8:c.183C>A	Pathogenic
7:44145522:C:G	*GCK*	p.Gly410Arg	ENST00000403799.8:c.1228G>C	Likely Pathogenic
7:44146531:G:C	*GCK*	p.His317Gln	ENST00000403799.8:c.951C>G	Likely Pathogenic
7:44145176:G:A	*GCK*	p.Ser453Leu	ENST00000403799.8:c.1358C>T	Likely Pathogenic
7:44145212:G:A	*GCK*	p.Ser441Leu	ENST00000403799.8:c.1322C>T	Likely Pathogenic
7:44153372:C:A	*GCK*	p.Arg46Met	ENST00000403799.8:c.137G>T	Pathogenic
7:44149859:CCTGCCAAGAAGCA:C	*GCK*	p.?	ENST00000403799.8:c.580-13_580-1del	Pathogenic
7:44150978:A:G	*GCK*	p.Val154Ala	ENST00000403799.8:c.461T>C	Likely Pathogenic
7:44149992:G:A	*GCK*	p.Arg186*	ENST00000403799.8:c.556C>T	Pathogenic
7:44153299:A:G	*GCK*	p.?	ENST00000403799.8:c.208+2T>C	Pathogenic
7:44146604:A:C	*GCK*	p.Ile293Arg	ENST00000403799.8:c.878T>G	Likely Pathogenic
7:44152318:G:A	*GCK*	p.Gln106*	ENST00000403799.8:c.316C>T	Pathogenic
7:44146619:C:T	*GCK*	p.?	ENST00000403799.8:c.864-1G>A	Pathogenic
7:44147765:G:A	*GCK*	p.Arg250Cys	ENST00000403799.8:c.748C>T	Pathogenic
7:44147741:C:T	*GCK*	p.Gly258Ser	ENST00000403799.8:c.772G>A	Likely Pathogenic
7:44145608:A:G	*GCK*	p.Met381Thr	ENST00000403799.8:c.1142T>C	Pathogenic
7:44149986:C:T	*GCK*	p.Ala188Thr	ENST00000403799.8:c.562G>A	Pathogenic
7:44150954:A:G	*GCK*	p.?	ENST00000403799.8:c.483+2T>C	Pathogenic
7:44145188:G:A	*GCK*	p.Ala449Val	ENST00000403799.8:c.1346C>T	Likely Pathogenic
7:44149779:G:T	*GCK*	p.Cys220*	ENST00000403799.8:c.660C>A	Pathogenic
7:44146463:C:A	*GCK*	p.Ser340Ile	ENST00000403799.8:c.1019G>T	Pathogenic
7:44145576:G:A	*GCK*	p.Arg392Cys	ENST00000403799.8:c.1174C>T	Pathogenic
7:44152268:CA:C	*GCK*	p.?	ENST00000403799.8:c.363+2del	Pathogenic
7:44150008:A:C	*GCK*	p.Asn180Lys	ENST00000403799.8:c.540T>G	Pathogenic
7:44146461:A:G	*GCK*	p.?	ENST00000403799.8:c.1019+2T>C	Pathogenic
7:44147720:C:T	*GCK*	p.Glu265Lys	ENST00000403799.8:c.793G>A	Pathogenic
7:44147690:G:C	*GCK*	p.Arg275Gly	ENST00000403799.8:c.823C>G	Likely Pathogenic
7:44145228:T:A	*GCK*	p.Ile436Phe	ENST00000403799.8:c.1306A>T	Likely Pathogenic

7:44150970:C:T	*GCK*	p.Glu157Lys	ENST00000403799.8:c.469G>A	Likely Pathogenic
7:44146619:C:G	*GCK*	p.?	ENST00000403799.8:c.864-1G>C	Pathogenic
7:44153351:G:A	*GCK*	p.Ala53Val	ENST00000403799.8:c.158C>T	Pathogenic
7:44151048:A:G	*GCK*	p.Ser131Pro	ENST00000403799.8:c.391T>C	Likely Pathogenic
7:44147732:C:T	*GCK*	p.Gly261Arg	ENST00000403799.8:c.781G>A	Pathogenic
7:44149968:C:T	*GCK*	p.?	ENST00000403799.8:c.579+1G>A	Pathogenic
7:44152275:TCAGCAGTG:T	*GCK*	p.Thr118Aspfs*8	ENST00000403799.8:c.351_358del	Pathogenic
12:120994313:G:GC	*HNF1A*	p.Pro289Alafs*28	ENST00000257555.11:c.863_864insC	Pathogenic
12:120993584:G:T	*HNF1A*	p.Lys197Asn	ENST00000257555.11:c.591G>T	Likely Pathogenic
12:120988909:GA:G	*HNF1A*	p.Asp135Valfs*20	ENST00000257555.11:c.404del	Pathogenic
12:120988898:G:A	*HNF1A*	p.Arg131Gln	ENST00000257555.11:c.392G>A	Pathogenic
12:120993591:C:T	*HNF1A*	p.Arg200Trp	ENST00000257555.11:c.598C>T	Pathogenic
12:120994262:G:A	*HNF1A*	p.Arg271Gln	ENST00000257555.11:c.812G>A	Pathogenic
12:120993519:G:A	*HNF1A*	p.?	ENST00000257555.11:c.527-1G>A	Pathogenic
12:120994267:AAAG:A	*HNF1A*	p.Glu275del	ENST00000257555.11:c.824_826del	Pathogenic
17:37731599:C:CA	*HNF1B*	p.Ser348Valfs*12	ENST00000617811.5:c.1040dup	Pathogenic
20:44424117:G:T	*HNF4A*	p.Arg309Leu	ENST00000316673.8:c.926G>T	Likely Pathogenic
20:44413696:G:A	*HNF4A*	p.Val108Ile	ENST00000316673.8:c.322G>A	Pathogenic
20:44406208:G:A	*HNF4A*	p.Arg67Gln	ENST00000316673.8:c.200G>A	Pathogenic
20:44424116:C:T	*HNF4A*	p.Arg309Cys	ENST00000316673.8:c.925C>T	Pathogenic
20:44419741:C:T	*HNF4A*	p.Arg231Trp	ENST00000316673.8:c.691C>T	Likely Pathogenic
20:44413709:G:A	*HNF4A*	p.Arg112Gln	ENST00000316673.8:c.335G>A	Pathogenic
20:44424117:G:A	*HNF4A*	p.Arg309His	ENST00000316673.8:c.926G>A	Likely Pathogenic
2:181678244:T:TG	*NEUROD1*	p.His206ProfsTer38	ENST00000295108.4:c.616dup	Pathogenic
13:27920208:C:CCGGCGCCGGAGTT	*PDX1*	p.Ser29GlyfsTer200	ENST00000381033.5:c.72_84dup	Pathogenic
13:27920410:C:CG	*PDX1*	p.Leu92AlafsTer133	ENST00000381033.5:c.273dup	Pathogenic
13:27920342:C:A	*PDX1*	p.Tyr68Ter	ENST00000381033.5:c.204C>A	Pathogenic
13:27920443:AG:A	*PDX1*	p.Gly103GlufsTer20	ENST00000381033.5:c.308del	Pathogenic
6:116882403:C:T	*RFX6*	p.Arg181Trp	ENST00000332958.3:c.541C>T	Likely Pathogenic
6:116916217:T:G	*RFX6*	p.Leu292*	ENST00000332958.3:c.875T>G	Pathogenic
6:116877348:C:T	*RFX6*	p.Gln25*	ENST00000332958.3:c.73C>T	Pathogenic
6:116919165:A:T	*RFX6*	p.Lys351*	ENST00000332958.3:c.1051A>T	Pathogenic
6:116880544:G:A	*RFX6*	p.Trp127*	ENST00000332958.3:c.381G>A	Pathogenic
6:116925596:G:T	*RFX6*	p.Gly608*	ENST00000332958.3:c.1822G>T	Pathogenic
6:116919243:C:T	*RFX6*	p.Arg377*	ENST00000332958.3:c.1129C>T	Pathogenic
6:116894011:CAA:C	*RFX6*	p.Lys198Argfs*26	ENST00000332958.3:c.593_594del	Pathogenic
6:116882404:G:A	*RFX6*	p.Arg181Gln	ENST00000332958.3:c.542G>A	Likely Pathogenic
6:116925497:CT:C	*RFX6*	p.Leu575Argfs*15	ENST00000332958.3:c.1724del	Pathogenic

Supplemental Table 3: Pathogenic variants from the US health system–based Geisinger Mycode cohort identified in this study.

Genomic Change	Gene	Protein Change	NucleotideChange	Classification
11:17413396:G:A	*ABCC8*	p.Arg826Trp	ENST00000302539.9:c.2476C>T	Pathogenic
7:44145602:G:A	*GCK*	p.Ser383Leu	ENST00000403799.8:c.1148C>T	Likely pathogenic
7:44146531:G:C	*GCK*	p.His317Gln	ENST00000403799.8:c.951C>G	Pathogenic
7:44146565:A:G	*GCK*	p.Leu306Pro	ENST00000403799.8:c.917T>C	Likely pathogenic
7:44147649:C:T	*GCK*	-	ENST00000403799.8:c.863+1G>A	Pathogenic
7:44147720:C:T	*GCK*	p.Glu265Lys	ENST00000403799.8:c.793G>A	Pathogenic
7:44147765:G:A	*GCK*	p.Arg250Cys	ENST00000403799.8:c.748C>T	Pathogenic
7:44149763:C:T	*GCK*	p.Val226Met	ENST00000403799.8:c.676G>A	Pathogenic
7:44149976:C:T	*GCK*	p.Arg191Gln	ENST00000403799.8:c.572G>A	Pathogenic
7:44149982:A:G	*GCK*	p.Ile189Thr	ENST00000403799.8:c.566T>C	Pathogenic
7:44150004:C:T	*GCK*	p.Val182Met	ENST00000403799.8:c.544G>A	Pathogenic
7:44152338:C:T	*GCK*	p.Trp99Ter	ENST00000403799.8:c.296G>A	Pathogenic
7:44153382:G:A	*GCK*	p.Arg43Cys	ENST00000403799.8:c.127C>T	Pathogenic
7:44153387:A:C	*GCK*	p.Met41Arg	ENST00000403799.8:c.122T>G	Likely pathogenic
7:44153411:A:G	*GCK*	p.Val33Ala	ENST00000403799.8:c.98T>C	Likely pathogenic
12:120988853:C:T	*HNF1A*	p.Ala116Val	ENST00000257555.11:c.347C>T	Pathogenic
12:120988897:C:T	*HNF1A*	p.Arg131Trp	ENST00000257555.11:c.391C>T	Pathogenic
12:120993601:G:A	*HNF1A*	p.Arg203His	ENST00000257555.11:c.608G>A	Pathogenic
12:120993619:C:A	*HNF1A*	p.Ala209Glu	ENST00000257555.11:c.626C>A	Likely pathogenic
20:44419783:C:T	*HNF4A*	p.Arg245Cys	ENST00000316673.9:c.733C>T	Likely Pathogenic
20:44428356:A:AT	*HNF4A*	p.Ala363CysfsTer35	ENST00000316673.9:c.1086dup	Pathogenic
13:27920184:GA:G	*PDX1*	p.Asp16AlafsTer107	ENST00000381033.5:c.47del	Pathogenic
13:27920230:GC:G	*PDX1*	p.Pro33LeufsTer90	ENST00000381033.5:c.98del	Pathogenic
13:27920254:G:GCCGCCAGCCC	*PDX1*	p.Pro45AlafsTer183	ENST00000381033.5:c.122_131dup	Pathogenic
13:27920308:T:TG	*PDX1*	p.Glu58GlyfsTer167	ENST00000381033.5:c.172dup	Pathogenic
13:27920342:C:A	*PDX1*	p.Tyr68Ter	ENST00000381033.5:c.204C>A	Pathogenic
6:116882403:C:T	*RFX6*	p.Arg181Trp	ENST00000332958.3:c.541C>T	Likely pathogenic
6:116919243:C:T	*RFX6*	p.Arg377Ter	ENST00000332958.3:c.1129C>T	Pathogenic
6:116919252:G:GT	*RFX6*	p.Ser381IlefsTer22	ENST00000332958.3:c.1139dup	Pathogenic
6:116922040:A:AGAT	*RFX6*	-	ENST00000332958.3:c.1329_1331dup	Pathogenic
6:116927295:TC:T	*RFX6*	p.Pro719LeufsTer54	ENST00000332958.3:c.2156del	Pathogenic

Supplementary Table 4: Comparison of cases by treatment subgroups in the UK Biobank.

Clinical Feature	Non-MODY insulin treated from diagnosis	Non-MODY NOT insulin treated from diagnosis	MODY Insulin treated from diagnosis	MODY NOT insulin treated from diagnosis	P-Value MODY Insulin treated from diagnosis vs MODY NOT insulin treated from diagnosis	P-Value MODY vs non-MODY (Insulin treated from diagnosis groups)	P-Value MODY vs non-MODY (NOT insulin treated from diagnosis groups)
**N**	1,176	23,705	5	126	NA	NA	NA
**Age at recruitment, y**	61.4 (55.8-65.2)	62.2 (56.8-66.1)	62.1 (55.8-64.8)	60.2 (55.1-64.9)	0.66	0.79	0.01
**Age at diabetes diagnosis, y**	50.5 (45.5-57)	56.5 (50.5-61.5)	58.5 (51.4-60.4)	54.1 (49.4-60.2)	0.82	0.3	0.003
**Females**	430 (36.6)	8,896 (37.5)	1 (20%)	62 (49.2%)	0.36	0.66	0.01
**European Ancestry**	996 (84.7)	19,990 (84.3)	5 (100%)	116 (92.1%)	1	1	0.014
**Parent with diabetes**	335 (28.5)	7,865 (33.2)	2 (40%)	54 (42.9%)	1	0.63	0.023
**BMI, kg/m^2**	29.4 (26.1-33.7)	30.9 (27.8-34.9)	28.9 (25.8-30.3)	27.7 (25.1-30.2)	0.73	0.46	2.29×10^{-13}
**HbA1c, mmol/mol**	59.8 (51-69.7)	51.1 (46.6-58.3)	61.9 (59.2-68.2)	50.5 (48.4-54.5)	0.007	0.73	0.46
**Triglycerides, mmol/l**	1.5 (1-2.4)	2 (1.4-2.8)	1.9 (1.7-2.7)	1.4 (1-2)	0.27	0.39	1.07×10^{-9}
**HDL, mmol/l**	1.2 (1-1.5)	1.1 (1-1.3)	1 (1-1.1)	1.4 (1.1-1.6)	0.031	0.15	3.02×10^{-8}
**LDL, mmol/l**	2.5 (2.1-3)	2.7 (2.2-3.3)	2.3 (2.3-2.7)	2.7 (2.3-3.5)	0.23	0.83	0.55

Supplementary Table 5: Comparison of cases by treatment subgroups in MyCode

Clinical Feature	Non-MODY insulin treated from diagnosis	Non-MODY NOT insulin treated from diagnosis	MODY Insulin treated from diagnosis	MODY NOT insulin treated from diagnosis	P-Value MODY Insulin treated from diagnosis vs MODY NOT insulin treated from diagnosis	P-Value MODY vs non-MODY (Insulin treated from diagnosis groups)	P-Value MODY vs non-MODY (NOT insulin treated from diagnosis groups)
N	3,988	22,577	8	34	NA	NA	NA
Age at recruitment, y	61.0 (53.7-68.5)	64.4 (56.2-71.3)	56.0 (53.4-63.4)	67.5 (52.8-73.1)	0.66	0.66	0.85
Age at diabetes diagnosis, y	56.7 (49.8-64.3)	58.1 (50.9-65.8)	50.7 (43.3-58.7)	58.8 (46.5-66.1)	0.26	0.26	0.64
Females	2045 (51.3)	11,426 (50.6)	2 (25%)	16 (47.1%)	0.46	0.34	0.81
European Ancestry	3633 (91.1)	21,610 (95.7)	8 (100%)	34 (100%)	1.0	2.34×10^{-9}	0.42
Parent with diabetes	1736 (43.5)	8535 (37.8)	5 (62.5%)	17 (50.0%)	0.38	2.63×10^{-7}	0.20
BMI, kg/m^2	34.0 (28.9-40.1)	34.1 (29.8-39.5)	34.7 (24.9-36.0)	28.7 (25.4-33.0)	0.086	0.76	2.8×10^{-4}
HbA1c, mmol/mol	71.6 (57.4-91.3)	54.1 (49.7-65.0)	63.4 (54.1-72.4)	54.1 (48.6-58.5)	0.72	0.0035	0.90
Triglycerides, mmol/l	1.8 (1-2.8)	1.9 (1.3-2.7)	1.9 (1.3-2.4)	1.7 (1.2-2.1)	1.0	0.85	0.35
HDL, mmol/l	1.1 (0.9-1.3)	1.1 (0.9-1.3)	1.3 (1.1-1.4)	1.3 (1.2-1.4)	1.0	0.84	0.055
LDL, mmol/l	2.6 (2.0-3.3)	2.7 (2.2-3.4)	1.5 (1.4-1.6)	3.0 (2.3-3.5)	1.0	0.11	0.26

Supplementary Table 6: Comparison of MODY cases with clinically diagnosed and screen detected non-MODY diabetes cases in UK Biobank.

Clinical Feature	MODY	Non-MODY only biomarker defined diabetes (screen detected)	Non-MODY clinically diagnosed (non-screen detected)	P-value MODY vs Non-MODY screen detected	P-value MODY vs Non-MODY clinically diagnosed
N	131	4,442	20,439	NA	NA
Age at recruitment, y	60.2 (55.1-64.9)	60.8 (54.7-65.3)	62.3 (57.2-66.2)	0.8	0.003
Age at diabetes diagnosis, y	54.5 (49.4-60.4)	60.8 (54.5-65.2)	55.5 (50.4-60.5)	$1.19\text{x}10^{-15}$	0.14
Females	63 (48.1%)	1869 (42.1%)	7457 (36.5%)	0.18	0.008
European Ancestry	121 (92.4%)	3634 (81.8%)	17352 (84.9%)	0.0011	0.014
Parent with diabetes	56 (42.7%)	1242 (28.0%)	6958 (34.0%)	0.00037	0.042
BMI, kg/m^2	27.9 (25.1-30.2)	31.1 (27.9-35.1)	30.8 (27.6-34.8)	$7.19\text{x}10^{-15}$	$5.91\text{x}10^{-13}$
HbA1c, mmol/mol	50.6 (48.5-54.8)	51.3 (49.3-57)	51.3 (45.3-59.3)	0.0021	0.91
Triglycerides, mmol/L	1.4 (1-2)	2.3 (1.6-3.2)	1.9 (1.3-2.7)	$6.72\text{x}10^{-17}$	$2.72\text{x}10^{-07}$
HDL, mmol/L	1.4 (1-1.6)	1.2 (1-1.4)	1.1 (1-1.3)	$2.58\text{x}10^{-5}$	$1.54\text{x}10^{-07}$
LDL, mmol/L	2.7 (2.3-3.5)	3.6 (2.9-4.2)	2.6 (2.2-3.1)	$1.27\text{x}10^{-16}$	0.013

N(%) for categorical data and median (interquartile range) for continuous data. * Below Bonferroni corrected threshold (p<0.004).

Supplementary Table 7: Comparison of MODY cases with clinically diagnosed and screen detected non-MODY diabetes cases in MyCode

Clinical Feature	MODY	Non-MODY only biomarker defined diabetes (screen detected)	Non-MODY clinically diagnosed (non-screen detected)	P-value MODY vs Non-MODY screen detected	P-value MODY vs Non-MODY clinically diagnosed
**N**	42	9,647	16,918	NA	NA
**Age at recruitment, y**	66.2 (52.8-72.7)	63.2 (55.7-70.7)	54.1 (49.7-65.0)	0.82	0.78
**Age at diabetes diagnosis, y**	57.9 (45.6-65.2)	57.2 (50.2-64.9)	58.3 (51.0-65.9)	0.56	0.25
**Females**	18 (42.9)	4863 (50.4%)	8608 (50.9%)	0.41	0.58
**European Ancestry**	42 (100)	9145 (94.8%)	16098 (95.2%)	0.24	0.27
**Parent with diabetes**	22 (52.4)	3951 (41.0%)	6320 (37.4%)	0.18	0.064
**BMI, kg/m2**	31.2 (27.4-34.0)	34.6 (30.2-40.2)	33.7 (29.3-39.3)	$1.99.19 \times 10^{-4}$*	2.95×10^{-3}*
**HbA1c, mmol/mol**	55.2 (48.6-67.2)	54.1 (48.6-67.2)	57.4 (50.8-71.6)	0.98	0.94
**Triglycerides, mmol/L**	1.7 (0.7-2.1)	1.9 (1.3-2.7)	1.7 (1.2-2.1)	0.28	0.48
**HDL, mmol/L**	1.3 (1.8-1.4)	1.1 (0.9-1.3)	1.3 (1.0-1.4)	0.019	0.15
**LDL, mmol/L**	3.0 (2.2-3.5)	2.7 (2.1-3.4)	2.7 (2.1-3.4)	0.54	0.59

N(%) for categorical data and median (interquartile range) for continuous data. * Below Bonferroni corrected threshold ($p<0.004$).
